# Features of an altered AMPK metabolic pathway in Gilbert’s Syndrome, and its role in metabolic health

**DOI:** 10.1038/srep30051

**Published:** 2016-07-21

**Authors:** Christine Mölzer, Marlies Wallner, Carina Kern, Anela Tosevska, Ursula Schwarz, Rene Zadnikar, Daniel Doberer, Rodrig Marculescu, Karl-Heinz Wagner

**Affiliations:** 1 University of Vienna, Faculty of Life Sciences, Department of Nutritional Sciences, Althanstraβe 14 (UZA2), 1090 Vienna, Austria; 2 University of Applied Sciences, FH JOANNEUM, Institute of Dietetics and Nutrition, Alte Poststraβe 149, 8020 Graz, Austria; 3 Medical University of Vienna, Center for Physiology and Pharmacology, Institute of Pharmacology, Währinger Straβe 13A, 1090 Vienna, Austria; 4 Medical University of Vienna, Clinical Institute of Laboratory Medicine, Vienna General Hospital, Währinger Gürtel 18-20, 1090 Vienna, Austria; 5 Medical University of Vienna, Department of Clinical Pharmacology, Vienna General Hospital, Währinger Gürtel 18-20, 1090 Vienna, Austria

## Abstract

Energy metabolism, involving the ATP-dependent AMPK-PgC-Ppar pathway impacts metabolic health immensely, in that its impairment can lead to obesity, giving rise to disease. Based on observations that individuals with Gilbert’s syndrome (GS; UGT1A1^*^28 promoter mutation) are generally lighter, leaner and healthier than controls, specific inter-group differences in the AMPK pathway regulation were explored. Therefore, a case-control study involving 120 fasted, healthy, age- and gender matched subjects with/without GS, was conducted. By utilising intra-cellular flow cytometry (next to assessing *AMPKα1* gene expression), levels of functioning proteins (phospho-AMPK α1/α2, PgC 1 α, Ppar α and γ) were measured in PBMCs (peripheral blood mononucleated cells). In GS individuals, rates of phospho-AMPK α1/α2, -Ppar α/γ and of PgC 1α were significantly higher, attesting to a boosted fasting response in this condition. In line with this finding, *AMPKα1* gene expression was equal between the groups, possibly stressing the post-translational importance of boosted fasting effects in GS. In reflection of an apparently improved health status, GS individuals had significantly lower BMI, glucose, insulin, C-peptide and triglyceride levels. Herewith, we propose a new theory to explain why individuals having GS are leaner and healthier, and are therefore less likely to contract metabolic diseases or die prematurely thereof.

The metabolism significantly impacts energy turnover on a cellular and systemic level. Under physiological conditions, energetic homeostasis is warranted by metabolising macro-nutrients (ATP refuelling) on the one hand, and by adaptive measures to compensate for energetic surplus on the other. In situations of chronic pathophysiological deregulation of key factors therein, metabolic complications including obesity can result. Secondary diseases are promoted, including type II diabetes mellitus (DM II), a condition that is among the most prominent pathological consequences of energetic misbalance^1^,^2^.

Healthy carriers of the UGT1A1^*^28 promoter mutation, that is characteristic for the benign condition of Gilbert’s syndrome (GS; *i.e.* M. Meulengracht), present with moderate unconjugated hyper-bilirubinaemia. The underlying polymorphism is characterised by an additional *–TA* repeat in the *TATA*-sequence of the *UGT1A1* promoter, to yield *(TA)*_*7*_*/(TA)*_*7*_ instead of *(TA)*_*6*_*/(TA)*_*6*_^3^. This missense mutation results in a reduced UGT1A1 enzyme function, leading to a decreased conjugation of bilirubin. Next to higher levels of unconjugated bilirubin (UCB) and in the absence of any other adverse symptoms, significantly lower body mass indices (BMI)^4^, improved glucose and lipid profiles^5^, and a resulting lower prevalence of DM II and of other chronic metabolic/inflammatory disorders^6^,^7^,^8^ have been reported specifically for this group. This imposed the assumption that crucial energetic switches such as AMPK (heterotrimeric AMP-activated ser/thr kinase), are likely positively affected in the condition of GS. Representing one of the most important energetic controllers and bottleneck of all energy consuming cellular processes, AMPK α1/α2 catalytic activity together with subsequent downstream metabolic effectors (PgC 1α, peroxisome proliferator-activated receptor gamma coactivator 1-alpha; Ppar α and γ, peroxisome proliferator-activated receptors α and γ; Sirt-1, sirtuin-1; FGF-21, fibroblast growth factor 21) were explored in terms of inter-group (GS and controls) differences in (activated) protein levels.

The core regulatory unit studied, AMPK, is a member of a metabolite sensing protein kinase family, that is present in all eukaryotes^9^, and retained in all cell types for regulating energy turnover. It is allosterically activated by increasing levels of ADP and AMP, and therefore considered primarily as a “fuelling-gauge” recognizing ATP depletion (as in fasting), limiting further energy consumption^10^. Together with a decline in ATP, upstream kinase activity determine AMPK’s activity through its phosphorylation status^11^,^12^. Active AMPK subsequently inactivates enzymes responsible for cholesterol-, fatty acid synthesis and gluconeogenesis. For years, this very mechanism has been exploited to routinely treat DM II, by using the anti-diabetic drug Metformin to increase AMPK phosphorylation, and ultimately improve glucose metabolism^13^,^14^.

Another important effect of AMPK activation includes the post-translational phosphorylation of PgC 1α, which is a positive regulator of energy consuming events such as oxidative processes (including mitogenesis and browning of adipose tissue), and adaptive thermogenesis. Its activity is furthermore fuelled in conditions of physical stress^15^, and is enhanced by the enzyme Sirt-1, another determinant of energy homeostasis^16^.

In immediate response to active PgC 1α and Sirt-1^16^, Ppars, an isotypic group of three (α, β/δ and γ^17^) nuclear receptor phospho-proteins^18^ and transcription factors^19^, are expressed. Means of their activation include phosphorylation through AMPK and ligand binding including fatty acids^20^. Ppars are specifically abundant in certain tissues including the liver, brain, muscle and cells of the immune system^21^. They occur ubiquitously in all cells^22^, as they control the expression of genes involved in adipogenesis and lipid metabolism. Therefore, Ppars are considered as crucial networkers of energy- and nutrient-catabolism^23^,^24^,^25^, which is why they are strongly implicated in the development and treatment of the metabolic syndrome^26^,^27^. Further associated with metabolic regulations, and upon Ppar α signaling, expression of FGF-21 takes place in the liver and adipose tissue^28^, from where it reaches the circulatory system. In animal studies, administration of this factor has been shown to positively impact metabolism in obese mice^29^ and diabetic monkeys^30^. Furthermore, serum FGF-21 has been suggested as a potential cardio-metabolic biomarker for humans^28^.

So far, there is no literature available on a possible role for the UGT1A1^*^28 polymorphism and/or elevated levels of UCB in these complex regulations. As yet, only one recent study^31^ points out a connection between circulating bilirubin (BR) and Ppar α, using an *in vitro* approach. This very report expands the field of known physiological bilirubin functions and activities, including antioxidant^32^, immune-modulating^33^ and signalling effects, the latter of which have been investigated in terms of Ppar activities in BR-treated mice. Results attest to an insulin-signalling effect of BR, ultimately modulating body weight, that appears to be in parts mediated through Ppar γ^26^.

Against this background, an observational case control study involving 120 healthy age- and gender-matched male and female subjects with and without GS, was conducted. The main aim was to further explain these striking metabolic differences, mainly reflecting in beneficial body composition, glucose- and lipid profile, as well as in apparently altered energetic regulations in response to fasting. To further explore particularities of metabolic regulation in GS, a molecular approach was used focusing on the AMPK pathway.

## Results

### Demographic and behavioural comparison between GS- and C subjects

Subjects between study groups did not significantly differ in terms of age distribution, or aspects of their lifestyles. As expected and crucial in terms of the study design, significant inter-group differences were found only for UCB and respective distribution of the number of TA-repeats (i.e. UGT1A1^*^28 genotype). These genotype distributions reflect what has been previously reported^34^,^35^. As for relevant differences in physical activity, male control (C) subjects (self-reportedly) were significantly more active than GS individuals (Table 1). There were no differences in parameters of liver health and iron status (AST, ALT, γ-GT, LDH, albumin, transferrin and ferritin) between GS and C individuals, as has been summarized by our group recently^36^.

### Comparison of metabolic parameters between GS- and C subjects

Biomarkers that were analysed in both study groups are summarized in Table 2 (including all subjects), Table 3 (for males only), and in Table 4 (for females only). Individual biomarkers were merged into groups, based on their respective effector-areas, namely energy-, carbohydrate- and lipid-metabolism.

Since exclusively healthy subjects were included in this study, all parameters reflecting metabolic health were within normal ranges throughout all groups (GS, C, males and females).

In the subsequent paragraphs, features of metabolic health are described, summarized for the entire study population, as well as split into gender groups.

#### Biomarkers associated with energy metabolism in GS- versus C subjects

##### All subjects

**Figure 1:**
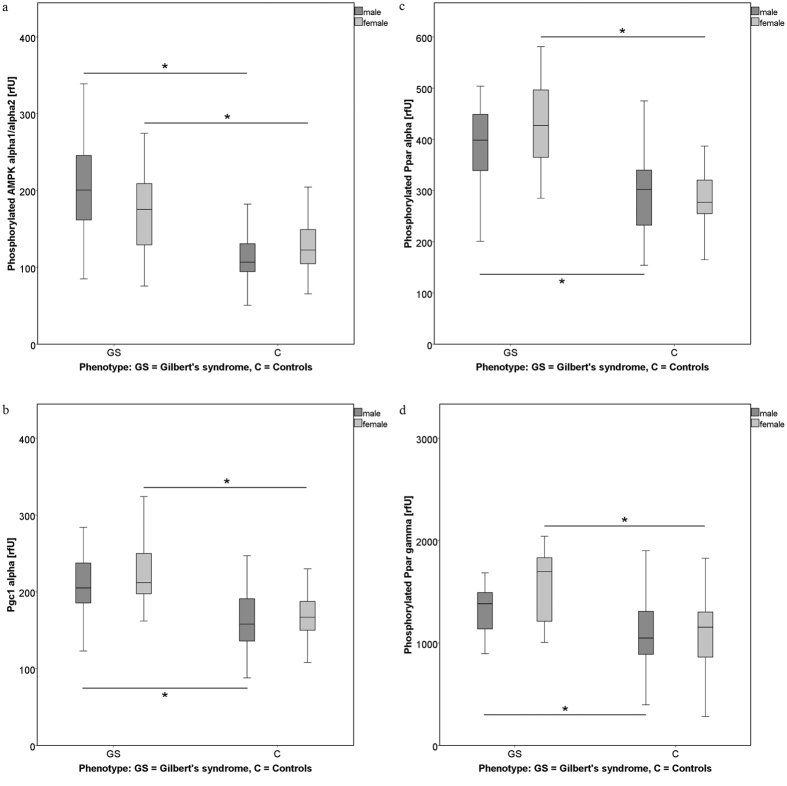
Comparison of measures of the AMPK pathway between the study groups (GS, C), including all subjects (male and female). Levels of (phosphorylated) proteins (AMPK α1/α2, Ppar α and γ, PgC 1α) were analysed using the method of flow cytometry. Data are expressed as relative fluorescence units [rfU], and compared between subjects with Gilbert’s syndrome (GS; UGT1A1*28 promoter mutation), and controls (C). * Indicates significant differences between groups (p ≤ 0.05). Medians can be found in Table 2. Abbreviations: pAMPK α1/α2: Phosphorylated 5′-AMP activated kinase; pPpar α: Phosphorylated peroxisome proliferator activated receptor alpha; pPpar γ: Phosphorylated peroxisome proliferator activated receptor gamma; PgC 1α: Peroxisome proliferator-activated receptor c coactivator 1.

Median phosphorylation/protein expression of AMPK α1/α2 and of associated downstream transcription factors (pPpar α, pPpar γ, PgC 1α; measured in PBMCs) was significantly higher in GS subjects versus controls (Fig. 1; p = 0.000). No group difference, however, was found in terms of *AMPKα1* gene expression (Table 2).

A trend towards higher FGF-21 serum concentration was found in GS relative to controls. No statistical difference was stated concerning Sirt-1 levels between groups, although values were slightly higher in GS as compared to controls.

Data on body composition differed between the groups, in that BMI levels were significantly lower in GS subjects relative to controls (p = 0.001), and lean body mass (LBM) was higher in GS, however, did not reach statistical significance.

##### Male subjects

Those significant differences in AMPK α1/α2 phosphorylation (and the downstream parameters pPpar α, pPpar γ, PgC 1α) found for both genders, were retained in males (p = 0.000, p = 0.003). Again, *AMPK1α* gene expression did not differ significantly between GS and C groups (Table 3).

Anthropometric measures were significantly different only in terms of BMI (p = 0.023), which was lower in the GS group. For males, no significant results were obtained for LBM.

##### Female subjects

Phosphorylation of AMPK α1/α2 and its downstream effectors (pPpar α, pPpar γ, PgC 1α) was significantly higher in female GS versus C subjects (p = 0.037, p = 0.000). In summary, this result is retained throughout the gender groups. For *AMPKα1* gene expression, again no significant results were found (Table 4).

In terms of body composition, both BMI and LBM differed significantly between the groups (p = 0.017, p = 0.011), in that BMI was lower and LBM was higher in female GS versus C.

#### Biomarkers associated with carbohydrate metabolism in GS- versus C subjects

##### All subjects

When considering the entire study population, fasting plasma glucose levels as well as concentrations of insulin and C-peptide, were significantly lower in the GS group, as compared to controls (p = 0.004, p = 0.001) (Table 2).

##### Male subjects

Again, as stated for the entire study group, fasting glucose levels were significantly lower in male GS versus C, as were insulin and C-peptide concentrations (p = 0.016, p = 0.009, p = 0.001) (Table 3).

##### Female subjects

Differences in parameters of glucose metabolism did not reach statistical significance between the study groups (Table 4).

#### Biomarkers associated with lipid metabolism in GS- versus C subjects

##### All subjects

Plasma TG levels were significantly lower in GS subjects (p = 0.045), LPA2 by trend was higher in GS. The remaining lipid parameters (as listed in Table 2) did not differ significantly between the groups (Table 2).

##### Male subjects

Those significant results reported above for both genders in terms of plasma TG and LPA2, were in retained in male subjects. LPA2 was again higher and TG levels were significantly lower in male GS (p = 0.048, p = 0.023). For the remaining parameters (as listed in Table 3), no significant results were obtained (Table 3).

##### Female subjects

As for the plasma lipid fractions, results for females are different to those presented for males. In female GS, LDL was lower by trend relative to C, as was the LDL/HDL ratio. Also Apo B trended to be lower in female GS as compared to controls (Table 4).

#### Inter-variable connection of metabolic parameters and the AMPK-pathway, is merely established *via* UCB and the underlying *UGT1A1* genotype (-TA repeats)

**Figure 2:**
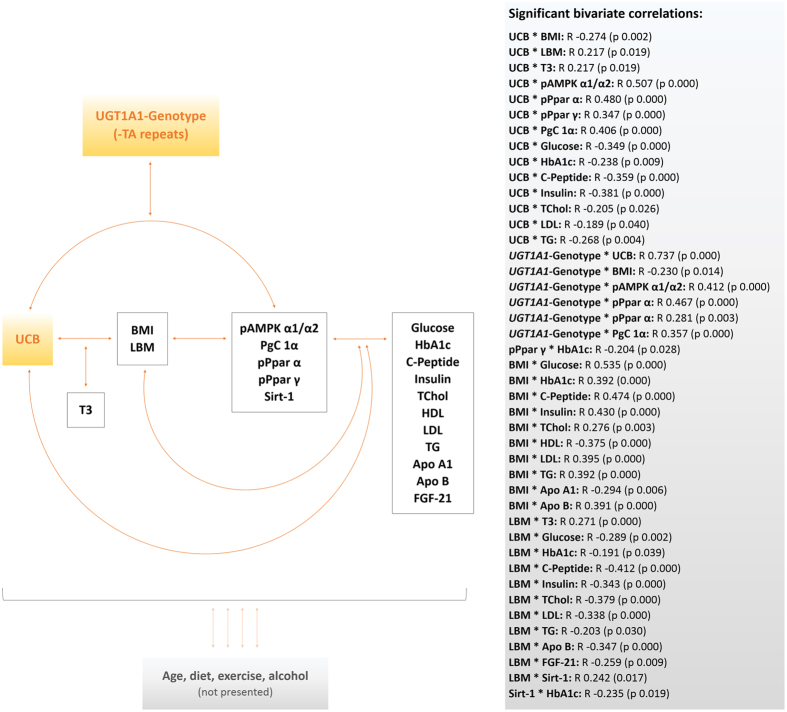
Graphical summary of inter-variable connections. Figure 2 illustrates statistical connections of variables of interest. Bivariate correlations were calculated for the entire study population using the model of Spearman’s rho. R coefficients and p-values (p ≤ 0.05; in brackets) are presented in the grey box and summarised in a graphical model. For ease of reading, lifestyle factors have not been incorporated in detail, and are therefore only abstracted (bottom of figure). Abbreviations: UCB: unconjugated bilirubin; *UGT1A1*: *UGT1A1* genotype; pAMPK α1/α2: Phosphorylated 5′-AMP activated kinase; pPpar α: Phosphorylated peroxisome proliferator activated receptor alpha; pPpar γ: Phosphorylated peroxisome proliferator activated receptor gamma; PgC 1α: Peroxisome proliferator-activated receptor c coactivator 1; Sirt-1: Sirtuin-1; FGF-21: Fibroblast growth factor 21; T3: Free triiodothyronine; BMI: Body mass index; LBM: Lean body mass; HbA1c: Glycated haemoglobin A1c; TChol: Total cholesterol; HDL: High density lipoprotein cholesterol; LDL: Low density lipoprotein cholesterol; TG: Triglyceride; LPA2: Lipoprotein A2; ApoA1: Apolipoprotein A1; ApoB: Apolipoprotein B.

For the reason of statistical validity and power, the entire dataset was analysed for inter-variable connections. A graphical summary of the correlations found is presented in Fig. 2, generating an idea as to how the parameters analysed could be networking on a physiological level. A detailed list of significant inter-variable associations, expressed as correlation coefficients (R) with corresponding p-values (in brackets), is provided in the figure.

**Figure 3:**
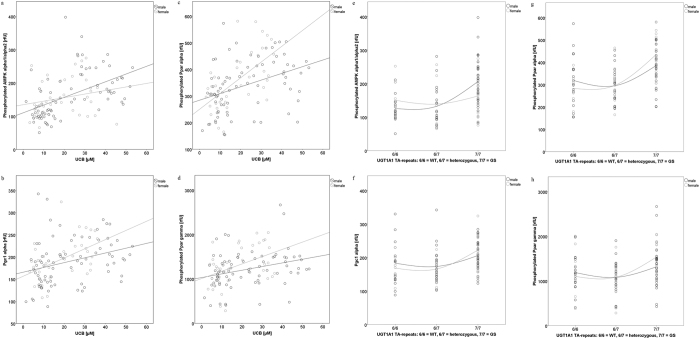
Correlations of UCB (**a–d**) and the UGT1A1 genotype (**e–h**), with measures of the AMPK pathway. Figure 3 illustrates gender-specific correlations of UCB and the UGT1A1 genotype with measures of the AMPK pathway. Bivariate correlations between UCB/*UGT1A1* genotype (-TA repeats: 6/6 controls, 6/7 heterozygous, 7/7 Gilbert’s syndrome) and measures of the AMPK pathway were calculated for each gender (m = male, f = female), using the model of Spearman’s rho. R coefficients and p-values (p ≤ 0.05; in brackets) are as follows: UCB * pAMPK α1/α2: m 0.594 (0.000); f 0.255 (0.122), UCB * PgC1 α: m 0.376 (0.001); f 0.467 (0.003), UCB * pPpar α: m 0.435 (0.000); f 0.575 (0.000), UCB * pPpar γ: m 0.354 (0.001); f 0.324 (0.047), ***UGT1A1*** * pAMPK α1/α2: m 0.541 (p 0.000); f 0.156 (p 0.362), ***UGT1A1*** * PgC1 α: m 0.265 (p 0.023); f 0.551 (p 0.001), ***UGT1A1*** * Ppar α: m 0.365 (p 0.002); f 0.661 (p 0.000), ***UGT1A1*** * Ppar γ: m 0.191 (p 0.023); f 0.435 (p 0.008). Abbreviations: UCB: unconjugated bilirubin; pAMPK α1/α2: Phosphorylated 5′-AMP activated kinase; pPpar α: Phosphorylated peroxisome proliferator activated receptor alpha; pPpar γ: Phosphorylated peroxisome proliferator activated receptor gamma; PgC 1α: Peroxisome proliferator-activated receptor c coactivator 1; WT: wild type (control subjects); GS: Gilbert’s syndrome.

To begin with UCB as the crucial determinant of the study design, and main feature of GS, strong correlations were found with pAMPK α1/α2, (R = 0.507, p = 0.000), and with downstream transcription factors (Fig. 2). A similar association was found when looking at the *UGT1A1* genotype, representing the underlying molecular background of GS (R = 0.412, p = 0.000). Gender-specific associations between UCB/*UGT1A1* and AMPK regulations, are visualized in Fig. 3. Interestingly, stronger correlations were generally found in women than in men.

Not surprisingly, UCB levels and *UGT1A1* genotype have been found to be strongly correlated (R = 0.731, p = 0.000). For these two specific features of GS (UCB and *UGT1A1* genotype), negative correlations were observed for a series of important lipid- and glucose biomarkers (as are listed in Fig. 2), emphasizing an improved metabolic state as UCB levels/TA-repeats increase.

The same negative connection applies to UCB/*UGT1A1* genotype and the anthropometric measure of BMI (R = −0.274, p = 0.002), whereas conversely, a positive association was found with LBM (R = 0.217, p = 0.019). Pursuing the interplay of anthropometric measures and other parameters, revealed connections of BMI and LBM with markers of lipid and carbohydrate metabolism (Fig. 2). Furthermore, an association of LBM with Sirt-1 was found (R = 0.242, p = 0.017), the latter of which statistically looping back to carbohydrate metabolism (HbA1c; R = −0.235, p = 0.019). HbA1c was also negatively associated with Ppar γ (R = −0.204, p = 0.028), one of the downstream effectors of the AMPK pathway, emphasizing its direct connection to energy- and carbohydrate metabolism (Fig. 2).

In summary, those correlations found point to close connections between characterising features of GS, body composition and an altered metabolic state in this condition, altogether having strong implications for macronutrient metabolism (carbohydrate and lipid), and importantly for energy turnover.

Heat maps visualizing correlated variables with respect to energy-, glucose- and lipid metabolism can be found on the online supplementary information (supplementary Figures S2 a, b, c).

#### It all starts with UCB: bilirubin has statistical explanatory power for AMPK pathway regulations and body composition

**Figure 4:**
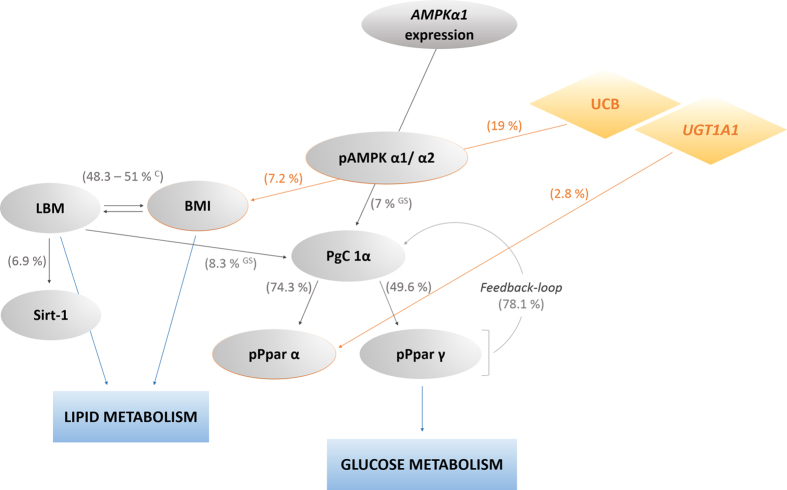
Abstract of inter-variable connection and dependence, based on regression analysis. Stepwise linear regression analysis was performed to assess inter-variable dependence and explanatory power (%), based on corrected R^2^ values and a p-value of ≤0.05 (Tables 5 and 6). ^C^effect found in control subjects only, ^GS^effect found in GS subjects only, (All remaining unspecified correlations are valid for both study groups.), Abbreviations: UCB: unconjugated bilirubin; *UGT1A1*: *UGT1A1* genotype; *AMPKα1:* 5′-AMP activated kinase α1 gene expression; pAMPK α1/α2: Phosphorylated 5′-AMP activated kinase; pPpar α: Phosphorylated peroxisome proliferator activated receptor alpha; pPpar γ: Phosphorylated peroxisome proliferator activated receptor gamma; PgC 1α: Peroxisome proliferator-activated receptor c coactivator 1; Sirt-1: Sirtuin-1; BMI: Body mass index; LBM: Lean body mass.

To further pursue inter-variable connections, and to explore possibilities as to how those entities studied could explain each other, stepwise linear regression models were generated. A graphical abstract of the most important findings, can be found in Fig. 4, where percentages (based on corrected R^2^ regression coefficients) specifying inter-variable explanatory power, are presented. Furthermore, tables summarising all relevant correlations that were found, are provided (Tables 5 and 6).

Most compelling, and in line with the findings from bivariate correlation analysis, UCB has noteworthy explanatory power for AMPK phosphorylation (pAMPK, 19%), and, although not as pronounced, for related pathway characteristics (pPpar α, 2.8%). Importantly, UCB furthermore connects the AMPK-pathway with body composition, by in parts explaining the variable BMI (7.2%), and possibly providing an important explanation for the significantly lower BMI stated in GS subjects, relative to controls (Table 1).

Interestingly, however not surprisingly, measures of body composition (BMI, LBM) appear to be merely linked to parameters of lipid metabolism, thereby likely explaining the improved lipid status determined in GS versus control-subjects in this (Tables 2), and previous studies^6^,^7^.

An entirely new inter-variable dependence was found, in that LBM had some explanatory power for Sirt-1 (6.9%), an important controller of metabolism with respect to ageing.

The measure of LBM was furthermore interlinked with PgC 1α, being the immediate activator of Ppar α and γ. This is clearly emphasized by its substantial explanatory power for the latter two (74.3% and 49.6%, respectively). Interestingly, there seems to be also an inverse correlation between these variables, suggesting a feedback-loop from Ppar α/γ to PgC 1α (78.1%).

As is known from the literature^37^,^38^, and newly reported here for the matrix of PBMCs, the AMPK pathway *via* its effectors Ppar α and γ, is ultimately linked to glucose metabolism, thereby likely explaining the relatively improved glucose metabolism generally determined in GS subjects (Tables 2, 3, 4). On a larger scale, this result provides a mechanism for the known low prevalence of type II diabetes among subjects having GS^8^.

Measures of lifestyle (physical activity, frequency of consuming specific foods) did not have significant further influence on variables of the AMPK pathway, as has been confirmed using regression analysis.

## Discussion

The aim of this study was to establish a theory to explain the compelling differences repeatedly found between GS and control subjects, concerning body composition and overall metabolic health. The AMPK pathway was the investigated model of choice, elegantly networking and determining features of energy- and macronutrient metabolism on a cellular and whole-body level. Specific findings of the study at hand are discussed and summarized in the following paragraphs. The obtained results provide robust evidence, that the energy- and macronutrient metabolic response to fasting are clearly boosted in GS. Accordingly, even though all subjects were metabolically healthy and within the reference ranges concerning their blood biochemistry parameters, several inter-group differences were found, confirming the improved metabolic health status of GS individuals. The relative extent to which these metabolic shifts are due to a direct effect of elevated UCB levels or based on a more complex genetic association with the UGT1A1*28 promoter mutation, remains to be clarified.

As stated in the materials and methods section, all 120 subjects were required to fast on the day before blood sampling (400 kcal restriction), as well as overnight for 16 (±1) hours. This is important to mention since serum UCB is known to rise in response to fasting, and was found to be the main determining factor of post-translational AMPK α1/α2 activation, as well as of BMI. This important feature of body composition was significantly lower in GS individuals.

### AMPK – it all starts with physical stress

The tight association of UCB and AMPK α1/α2 activity is not surprising and was expected, since both effectors are triggered by fasting leading to a drop in ATP, and by other physical stressors including exercise. On a molecular level, these common influencing variables emphasize a potential molecular cross-talk, thereby connecting GS genetics with AMPK α1/α2 activation. The importance of an altered fasting response specifically in GS, is further highlighted by the fact that levels of *AMPKα1* gene expression were equal between the groups. With reference to the influencing factor of physical activity, control subjects (self-reportedly) more frequently engaged in physical activity. In theory this study group (C) should have thus benefitted from the various health-benefits known to arise from exercising, and AMPK activity should have been higher. However, the obtained results reported an opposing trend, in that AMPK phosphorylation was significantly higher in GS versus controls. Therefore AMPK α1/α2 was more re-/active, with significantly improved parameters of metabolic health, including glucose, C-peptide, insulin and TG in GS subjects. Interestingly the parameter of TG in fact was found to be the “universal” statistical connector and determinant of the pathways explored, in that it had explanatory powers for C-peptide, insulin, and all fractions of cholesterol (TChol, HDL, LDL).

### AMPK from a different angle – metabolic interplay in GS

With reference to the observed phenomenon of lower plasma lipid fractions, it is well established that Ppar α and γ trigger pathways that are involved in lipogenesis and lipid storage^39^,^40^. When activated through ligand-binding or phosphorylation (*e. g.* through AMPK), these pathways lead to body-wide redistribution of fat, consequently lowering TG levels, and thereby improving insulin sensitivity. This effect was clearly present in male GS individuals, expressed through significantly lower TG levels, along with slightly lower TChol, and an improved glucose metabolism. This confirms the above role for Ppars as ultimate regulators of lipid metabolism (ultimately influencing that of glucose), and implies an even more pronounced metabolic effect in GS individuals, likely based on their more re-/active AMPK pathway. These important results readily connect to the low prevalence of metabolic diseases previously stated for GS individuals^6^,^7^.

The significantly lower TChol levels previously stated for GS individuals^5^, and the slightly lower TChol levels reported for GS individuals in this study, could be predicated once more on this group’s increased AMPK activity. AMPK has a known post-translational deactivating effect on HMG-CoA reductase (3-hydroxy-3-methyl-glutaryl-CoA reductase; not measured in this study), which is the rate-limiting enzyme in cholesterol synthesis^41^. This may be hypothesised to contribute to the slightly lower TChol levels in the GS group.

With reference to aspects of glucose regulation, a statistical association of Ppar γ activity (together with glucose), and the long term glucose parameter HbA1c was found. This is in agreement with results from animal studies in which an indirect BR-mediated insulin effect through the direct Ppar γ agonist HO-1^26^, and an insulin-sensitizing activity of BR based on anti-inflammation^42^ have been reported. The overall effects and proposed underlying mechanisms were similarly found in the present study, in that GS individuals (like BR-treated mice in the above studies), were generally lighter, leaner and as mentioned, had improved glucose parameters relative to controls.

### Key players in body composition – UCB, Ppars and anti-inflammation

Excess body mass is a known crucial regulator of glucose homeostasis, lipid metabolism^43^,^44^ and inflammation^45^. An increased fat mass triggers inflammation, involving the production and release of TNFα and IL-6^45^. Inversely, the comparably higher LBM that was determined in female GS individuals, as well as the lower BMI reported for all GS individuals, both would have an easing impact on inflammation. In the present study, it is evident that entities of metabolic health are apparently influenced to a large extent by BMI. This marker was in part explained by UCB levels, thereby connecting AMPK activity with body composition (Fig. 4). C-reactive protein (CRP), as well as TNFα and IL-6 were in fact (significantly) lower in GS individuals as compared to controls (results presented in ref. 36). This emphasizes the above hypothesis of a possible involvement of BR (UCB in the present study), in lowering inflammation through an increase in energy turnover (AMPK phosphorylation), and provides a link to improved body composition. Considering the fact that all study participants were free of (inflammatory) diseases, this result is particularly remarkable.

With reference to body composition another interesting observation was made in that LBM had explanatory power for Sirt-1, a known controller of metabolism with specific relevance to ageing. This result could further bridge the gap towards explaining the epidemiological evidence for longevity in GS, which has been experimentally explored recently^36^.

More detailed statistical analyses into body composition and its connection to energy metabolism revealed an interesting gender-specific effect, which to date cannot be definitively explained. It is, however, possibly based on the gender-specific difference in oestrogen levels, that are known to influence energy metabolic pathways^46^,^47^. As mentioned, LBM was significantly higher in GS individuals (relative to controls) only in females, and the beneficial difference in BMI between the two female groups was more pronounced as compared to that between the male groups (GS versus C). Ultimately connecting these results to energy turnover, they are readily confirmed by the generally stronger correlations between the AMPK pathway and increasing –TA repeats and UCB levels, found in women as compared to men (Fig. 3a,b). These observations are particularly remarkable in view of the relatively smaller female versus male group sizes.

## Summary

In conclusion, the AMPK pathway not only is a master regulator of (energy) metabolism and main crossroad of various pathways, it furthermore seems to be a powerful switch that in GS more readily reacts to fasting, possibly leading to an increased energy turnover in this condition.

In this study, (i) not only those beneficial metabolic features were confirmed that had been established previously for GS individuals, but (ii) also the new finding of an apparently boosted AMPK pathway in GS in response to fasting, was presented for the first time. To this end, it cannot be estimated to which extent a potential increase in energy turnover in GS individuals is based on adaptive thermo- and mitogenesis, and possibly adipose tissue browning.

However, our findings expand and complement data previously obtained from animal studies^26^,^42^, and propose a precise connection point to pursue future investigations into the molecular background and particularities of metabolic regulation in GS. Specific approaches could include immune-precipitation analyses to assess potential direct binding of BR as Ppar agonist, or microarray/genome wide association studies (GWAS) to screen for SNP-SNP associations or for SNP-interactions with certain phenotypic characteristics and aspects of metabolism in GS.

## Materials and Methods

### Subjects and study design

This study (abbreviated “BiliHealth”) was designed as an observational case-control study, at a single centre in Vienna, Austria. The study was performed at the Department of Clinical Pharmacology at Vienna General Hospital, and subjects were recruited between June 2014 and January 2015, by direct advertising (bulletin boards, posters and flyers) and from the department’s subject database.

One hundred twenty-eight (128) healthy subjects between 20 and 80 years of age were initially recruited from the general Austrian population. Eight thereof, had to be excluded for medical reasons. Exclusion criteria included smoking, excess drinking, routine intake of medications and nutritional supplements, pregnancy, acute and chronic (inflammatory/metabolic) diseases, liver diseases, present or past neoplasia and organ transplants. After providing their signed written consent form, each subject completed an initial health check-up (fasting blood biochemistry including levels of unconjugated bilirubin (UCB) and liver enzymes, blood pressure, body weight/-height, questionnaires).

A total of 80 males and 40 females completed the study. This gender distribution is representative of the occurrence of GS in the general population^48^. All subjects were age- and gender-matched, and study group allocation (GS, C) was based on the subjects’ respective fasting serum UCB concentrations (</≥17.1 μM)^48^, that had been analysed using HPLC. For the most part, subjects with GS (in contrast to C) showed visible signs of mild jaundice, reflecting in a yellowish pigmentation of the skin and the conjunctival membranes over the sclerae. Liver parameters and parameters of haemolysis were within the normal ranges. Participants were furthermore allocated to age groups (</≥35 years of age). For a graphical summary of the study design refer to supplementary Figure S1.

For the purpose of diagnosing GS, all subjects of both study groups were required to fast on the day before participating in the study, and therefore had to follow a 400 kcal fasting protocol^4^,^49^. Furthermore, a complete overnight fast of 16 (±1) hours was required, before the day of blood sampling.

Characteristics of the study population, including age distribution, UCB levels and aspects of lifestyle, are summarized in Table 1.

### Ethics

This study was approved by the Ethics Commission of the Medical University of Vienna (No. 1164/2014), and was conducted in accordance with the approved guidelines by the Declaration of Helsinki.

### Blood biochemistry (whole blood, plasma, serum)

For each subject, fasting blood samples were collected on a single occasion (baseline), no longer than two weeks from the entry health check-up. Samples were drawn by venepuncture into EDTA, Li-Heparin and serum tubes (K_2_EDTA, Li-Heparin and Z Serum Sep, respectively). Samples were cooled and protected from light until being analysed or aliquoted. Aliquots were stored at −80 °C until further analysis.

Besides UCB, liver enzymes (aspartate aminotransferase, AST; alanine transaminase, ALT; gamma-glutamyl trans peptidase, γ-GT; lactate dehydrogenase, LDH), ferritin, transferrin, hormones (thyroid stimulating hormone, TSH; triiodothyronine, T3; thyroxin, T4) and a range of lipid parameters (total cholesterol, TChol; high density lipoprotein, HDL; low density lipoprotein, LDL; triglycerides, TG; ApoA1, apolipoprotein A1; ApoB, apolipoprotein B; lipoprotein A2, LPA2) were automatically analysed in the routine central laboratories of the Vienna General Hospital (Olympus 5400 clinical chemistry analysers, Beckman Coulter). All parameters were measured on the day of blood sampling.

### UCB measurement (HPLC) in serum

For a detailed analysis of UCB (isomers), the method of HPLC was applied (after)^50^, as had been used and published by our group^4^,^51^ and others^52^ previously. Briefly, fasting serum samples (stored light-protected in amber vials) were diluted in isocratic mobile phase (methanol, water, *n*-dioctylamine and acetic acid) and centrifuged. Supernatants were run on a chromatograph (Merck, Hitachi, LaChrom), equipped with a photodiode array detector (PDA, Shimadzu) and a Fortis C18 HPLC column (4.6 × 150 mm, 3 μm), with a Phenomenex C18 HPLC guard column (4 × 3 mm). Sample preparation and analysis followed the previously published protocol^4^. Unconjugated bilirubin (Frontier Scientific Europe, Carnforth, Lancashire, UK) served as an external standard/quality control. As an internal standard, a reference serum sample was run in each analysis.

### *UGT1A1* Genotyping (-TA repeats in UGT1A1*28 promoter region)

For *UGT1A1* genotyping purposes, DNA was extracted from whole blood, using QIAsymphony SP automated system with QIAsymphony DSP DNA Midi Kit (QIAGEN), as instructed.

Analyses were performed as described elsewhere^53^. Primers and probes were used as 10 μM working solutions. LightCycler FastStart DNA Master HybProbe Mix (Roche) was used on a LightCycler 480 Instrument II (Roche). Alleles were determined according to the melting curves obtained.

### Anthropometric measurements

Standing height (subjects without shoes and in relaxed upright position) was measured with a commercial stadiometer, to the nearest 0.5 cm. Body mass (subjects barefooted and lightly dressed) was assessed to the nearest 0.1 kg, using digital scales. The body mass index (BMI) was calculated following the equation BMI = body mass [kg]/(body height [m^2^])^2^. To determine body composition, Bioelectric Impedance Analysis (BIA) was used, providing reliable data of body composition^54^, and was performed in the mornings of the study days, using a BIA Analyser 2000-S (Data-Input GmbH, Darmstadt, Germany).

### Lifestyle assessment of subjects

All participants were required to answer questions about their lifestyle, including (everyday) activity, exercise/training, drinking and eating habits. For this purpose, food frequency and lifestyle questionnaires were completed by each subject.

Indices of food intake were calculated for the reported weekly frequency of health food, snack food and red meat as well as alcohol consumption, and statistically analysed. “Health foods” included data on foods rich in vitamins, antioxidants, unsaturated fatty acids and fibres; “snack foods” referred to fatty and sugary energy-dense snacks; “red meat” included specifications on intake of red meat and meat products; “alcohol” referred to weekly alcoholic beverage consumption.

For details on weekly frequency of bodily activity, indices on overall activity, endurance exercise and resistance exercise were calculated. “Overall activity” included climbing stairs and walking; “endurance exercise” referred to frequency of at least 30 min bouts of endurance training; “resistance training” included reported frequency of resistance exercise (using own body weight and/or weights) per week.

### Flow cytometric (FACS) analyses of pAMPK α1/α2, PgC1 alpha, pPpar alpha and –gamma in PBMCs

Active (phosphorylated) intracellular protein concentrations were measured in peripheral blood mononucleated cells (PBMCs). Cells were extracted from EDTA whole blood immediately after blood samplings. Density gradient centrifugation using separation tubes (Leucosep^TM^, Greiner bio one GmbH, Austria) was applied as instructed. Following isolation, cells were washed twice with ice-cold PBS. Cell count and viability were assessed using the trypan blue exclusion assay on an automated cell counter (Countess^TM^, Life Technologies). For short-term storage, cells were aliquoted in freezing medium (FBS + 10% DMSO) and gradually cooled (1 °C/min) to −80 °C, using the CoolCell^TM^ system (Biozym).

All FACS analyses were completed on a four-channel FACS Calibur^TM^ flow cytometer (BD, Europe). Signal compensation (using Calibrite^TM^ beads and FACS Comp software, BD) was successfully completed prior to each experimental run.

PBMCs were thawed at 37 °C and washed twice with cold PBS (3000 g, 5 min). Cell count per test tube was adjusted to 250.000. After washing, cells were fixed (1% formalin, 10 min, RT), washed again, and permeabilised (70% ice-cold ethanol, 10 min, on ice). Following another washing step, cell pellets were suspended in staining-buffer (1% BSA, 0.02% Na-acide in PBS), and stained with respective antibodies (30 min, on ice; where applicable, the same is true for secondary antibody). All antibodies used had been titrated before use. Samples were run twice as independent duplicates, relative to respective negative/isotype controls. Antibodies were duplexed, so that cross-channel signal interference could be excluded. The antibody set-up used was as follows: anti-phospho-AMPK: rabbit anti-human monoclonal to AMPK α1 (phos-T183) and AMPK α2 (phos-T172), (ab133448, Abcam); secondary antibody to phospho-AMPK: goat anti-rabbit IgG H & L AlexaFluor 488, (ab150077, Abcam); anti-PgC 1α: rabbit anti-human polyclonal to PgC 1α, PE-labelled, (orb124814, Biorbyt); anti-phospho-Ppar α: rabbit anti-human polyclonal to Ppar α (phos-Ser12), FITC-labelled, (bs-4055R-FITC, Bioss); anti-phospho-Ppar γ: rabbit anti-human polyclonal to Ppar γ (phos-Ser112), AlexaFluor 647 labelled, (bs-3737R-A647, Bioss). Fluorescence signals (relative fluorescence units, rfU) were detected and recorded in the respective channels, and compared between study groups.

#### RNA extraction, cDNA synthesis and qPCR of *AMPK1a* gene expression

RNA was extracted from PBMCs using Qiagen RNeasy^®^ Mini Kit, as instructed by the manufacturer, and using the QIAcube automated system. Total RNA concentration and quality were estimated using NanoDrop® ND-1000 (Thermo Scientific). The synthesis of cDNA from RNA was performed using the High-Capacity cDNA Reverse Transcription Kit with RNase Inhibitor 1000 Reactions (Applied Biosystems) on a Biometra thermocycler. Concentration and quality of cDNA first strand was determined by NanoDrop 2000c spectrophotometer.

For qPCR 10ng cDNA samples were used on commercially available TaqMan assays (Life Technologies) as single-plex reactions. The TaqMan assay for PRKAA1 (assay Hs01562315_m1, FAM-MGB-labelled) gene was used according to manufacturer instructions. ACTB (assay Hs99999903_m1) and GAPDH (Hs99999905_m1) were used as endogenous controls.

The assays were performed using TaqMan Universal PCR master mix on a QuantStudio™ 6 Flex Real-Time PCR System (Thermo Fisher), on a 384-well block. All samples were run on the same 384-well plate in a single run, to avoid inter-plate variations. All samples were run in triplicate, and samples with a standard deviation higher than 0.5 Ct-units were excluded from further analyses. Relative quantification was performed using the RQ (Relative Quantification) feature on the Thermo Fisher Cloud qPCR analysis software with ACTB and GAPDH as endogenous controls and a pooled cDNA sample as a reference sample.

### ELISA measurement of FGF-21 and Sirt-1 protein levels in serum

Fibroblast growth factor-21 (FGF-21) as well as Sirtuin-1 (Sirt-1) were measured in serum using respective ELISA kits (FGF-21 Human ELISA Kit, ab125966; Human SIRT1 ELISA Kit SimpleStep, ab171573; both Abcam), and following manufacturer instructions. Plate assays were run in a 96-well plate format, using a BMG FLUOstar OPTIMA microplate reader (BMG LABTECH GmbH), set to absorbance mode (450 nm). All samples and external standards were run in duplicate.

### Statistical analyses

Statistics were completed using IBM SPSS 21 [IBM Corp. Released 2012. IBM SPSS Statistics for Windows, Version 21.0. Armonk, NY: IBM Corp.], as well as COVAIN toolbox for MATLAB [MATLAB and Statistics Toolbox Release 2015a, The MathWorks, Inc., Natick, Massachusetts, United States.], for generating correlation heat maps.

Data distribution was checked using Kolmogorov-Smirnov (K-S test) and histograms. For comparison of means (for parametric data), ANOVA was used, for comparing medians or ranks (for non-parametric data), Mann-Whitney-U-test was selected. Data are summarized as is appropriate according to their respective distribution. For parametric data, means ± sd (standard deviation), for non-parametric variables, medians ± IQR (inter-quartile range) are presented. Bivariate correlations were modelled using Spearman correlation (Spearman’s rho). Correlation coefficients (R) and p-values are presented. Regressions were calculated by applying the model of stepwise linear regression. Corrected R2 coefficients and p-values are presented. For all statistical measures, the level of significance was set to be 5% (p ≤ 0.05).

## Additional Information

**How to cite this article**: Mölzer, C. *et al*. Features of an altered AMPK metabolic pathway in Gilbert’s Syndrome, and its role in metabolic health. *Sci. Rep.* **6**, 30051; doi: 10.1038/srep30051 (2016).

## Supplementary Material

Supplementary Information

## Figures and Tables

**Table 1:** Demographical and behavioural description of the BiliHealth study population (all subjects).

Variables	Males		*p-value*	Females		*p-value*
	*GS*	*C*		*GS*	*C*	
Subjects [n]	40	40	*1.000*	20	20	*1.000*
Median age [yrs]^	30 (19)	31 (20)	*0.965*	40 (20)	40 (18)	*0.766*
Subjects aged </≥ 35 yrs [n/n]	24/16	24/16	*1.000*	9/11	9/11	*1.000*
Age of subjects </≥ 35 yrs^	26.5 (6.0)/45.5 (15.0)	27.0 (6.0)/45.5 (14.0)		29 (5)/48 (19)	29 (5)/46 (14)	
UCB concentration [μM]°	35.3 (±10.4)	9.7 (±3.5)	0.000*	28.9 (±6.8)	8.4 (±3.1)	0.000*
UGT1A1*28 genotype/-TA repeats [% 7_7/6_7/6_6]^□^	86.8/10.5/2.5	5.3/50/44.7	0.000*	94.7/5.3/0	5.3/45/45	0.000*
Health food consumption [times/week]°	28.6 (±9.3)	24.5 (±13.2)	*0.153*	32.0 (±8.0)	31.0 (±7.0)	*0.611*
Snack food consumption [times/week]^	10.5 (9.3)	10.0 (7.0)	*0.941*	9.0 (11.5)	13 (11.5)	*0.876*
Red meat consumption [times/week]^	3.0 (6.5)	3.0 (6.0)	*0.891*	0 (3)	3 (4)	*0.103*
Alcoholic drinks consumption [times/week]^	1 (3)	0 (2)	*0.419*	0 (4)	1 (3)	*0.945*
Overall activity [times/week]^	5.0 (3.5)	6.5 (5.5)	0.025*	6.0 (3.5)	4.0 (4.1)	*0.069*^*T*^
Endurance exercise [times/week]^	2 (2)	3 (5)	0.003*	4.0 (3.0)	2.0 (2.2)	*0.074*^*T*^
Resistance exercise [times/week]^	1 (2)	1 (2)	*0.908*	0.3 (2.0)	0.0 (1.8)	*0.667*

(Table 1 provides a comparative (GS versus C) demographical and behavioural description of all subjects of the BiliHealth study.

Based on data distribution, means° or medians^ are presented. For parametric data mean ± sd, for non-parametric distribution, medians and IQR (inter-quartile range) are given. P-values of ≤0.05^*^ indicate significant differences; trends are reflected by p ≤ 0.1^T^.

^◽^Insertion of additional –TA repeats in the UGT1A1^*^28 promoter region; 7_7: Gilbert’s syndrome, 6_7: heterozygous individuals, 6_6: wild type.

Abbreviations

UCB: unconjugated bilirubin; UGT1A1-genotype: UDP glucuronosyltransferase 1A1 genotype.

**Table 2:** Energy-, carbohydrate and lipid metabolic biomarkers of all subjects of the BiliHealth study.

	Variable	Mean° (±sd)/median^ (IQR)		p-value^*/T^
		*GS*	*C*	
*Biomarkers of energy metabolism*	pAMPK α1/α2 [rfU]^	187 (94) n = 58	112 (43) n = 56	0.000*
	pPpar α [rfU]°	400 (±88) n = 57	292 (±80) n = 58	0.000*
	pPpar γ [rfU]°	1401 (±398) n = 58	1086 (±378) n = 58	0.000*
	PgC 1α [rfU]^	206 (56) n = 58	165 (55) n = 58	0.000*
	AMPK α1 expr. [RQ]^	0.76 (0.25) n = 60	0.80 (0.25) n = 60	*0.592*
	Sirt-1 [ng/mL]^	3.16 (1.64) n = 47	2.92 (1.18) n = 52	*0.491*
	FGF-21 [μg/mL]^	0.35 (0.43) n = 52	0.10 (0.43) n = 51	*0.086*^*T*^
	TSH [μU/mL]^	1.8 (1.2) n = 57	2.0 (1.2) n = 59	*0.404*
	T3 [pg/mL]°	3.24 (±0.41) n = 58	3.22 (±0.39) n = 59	*0.844*
	T4 [ng/dL]^	1.29 (0.20) n = 58	1.26 (0.20) n = 59	*0.105*
	BMI [kg/m^2^]°	22.8 (±3.0) n = 60	25.4 (±4.9) n = 60	0.001*
	LBM [%]^	78.5 (8) n = 59	76.2 (13) n = 58	*0.105*
*Biomarkers of carbohydrate metabolism*	Glucose [mg/dL]^	81 (7) n = 59	86 (11) n = 60	0.004*
	HbA1c [%]^	5.0 (0.4) n = 60	5.1 (0.5) n = 60	*0.116*
	C-Peptide [ng/mL]^	1.2 (0.6) n = 58	1.4 (1) n = 59	0.004*
	Insulin [μU/mL]^	4.1 (2.5) n = 55	5.9 (4.7) n = 59	0.001*
*Biomarkers of lipid metabolism*	TChol [mg/dL]^	171 (47) n = 58	182 (67) n = 60	*0.112*
	HDL Chol [mg/dL]^	62 (25) n = 58	63 (27) n = 60	*0.726*
	LDL Chol [mg/dL]^	93 (35) n = 58	100 (64) n = 60	*0.112*
	LDL/HDL ratio^	1.4 (0.7) n = 58	1.6 (1.2) n = 60	*0.162*
	TG [mg/dL]^	73 (31) n = 57	85 (50) n = 60	0.045*
	LPA2 [mg/dL]^	17 (21) n = 54	13 (18) n = 55	*0.082*^*T*^
	ApoA1 [mg/dL]°	149 (±27) n = 60	152 (±30) n = 60	*0.638*
	ApoB [mg/dL]°	83 (±24) n = 60	90 (±28) n = 60	*0.194*
	Apo B/A1 ratio^	0.5 (0.2) n = 59	0.6 (0.3) n = 60	*0.409*

(Table 2 summarises and compares (GS versus C) biomarkers of energy-, carbohydrate and lipid metabolism of all subjects of the BiliHealth study.

Values are specified as applies according to distribution of data. For parametric variables, means° ± sd are shown, for non-parametric data, medians^ (50^th^ percentiles) and inter-quartile range (IQR) are displayed. Comparison of means for parametric data or of ranks (for non-parametric data) was completed using independent samples t-test or Mann-Whitney-U-test. P-value*: significant on a 5% level of significance; p-value^T^: trend on a 10% level of trend

Abbreviations: GS: Gilbert’s syndrome; C: Controls; pAMPK α1/α2: Phosphorylated 5′-AMP activated kinase; pPpar α: Phosphorylated peroxisome proliferator activated receptor alpha; pPpar γ: Phosphorylated peroxisome proliferator activated receptor gamma; PgC 1α: Peroxisome proliferator-activated receptor c coactivator 1; AMPK α1 expr.: AMPK gene expression as relative quantification, RQ to cDNA pool; Sirt-1: Sirtuin-1; FGF-21: Fibroblast growth factor 21; TSH: Thyroid stimulating hormone; T3: Free triiodothyronine; T4: Free thyroxine; BMI: Body mass index; LBM: Lean body mass; HbA1c: Glycated haemoglobin A1c; TChol: Total cholesterol; HDL: High density lipoprotein cholesterol; LDL: Low density lipoprotein cholesterol; TG: Triglyceride; LPA2: Lipoprotein A2; ApoA1: Apolipoprotein A1; ApoB: Apolipoprotein B.

**Table 3:** Energy-, carbohydrate and lipid metabolic biomarkers of male subjects of the BiliHealth study.

MALES	Variable	Mean° (±sd)/median^ (IQR)		p-value^*/T^
		*GS*	*C*	
*Biomarkers of energy metabolism*	pAMPK α1/α2 [rfU]^	201 (91) n = 39	107 (39) n = 37	0.000*
	pPpar α [rfU]°	385 (±84) n = 38	296 (±90) n = 39	0.000*
	pPpar γ [rfU]^	1384 (365) n = 39	1049 (438) n = 39	0.003*
	PgC 1α [rfU]^	205 (53) n = 39	158 (56) n = 39	0.000*
	AMPK α1 expr. [RQ]^	0.76 (0.24) n = 40	0.78 (0.25) n = 40	*0.729*
	Sirt-1 [ng/mL]^	3.26 (1.66) n = 31	3.24 (1.77) n = 34	*0.887*
	FGF-21 [μg/mL]^	0.33 (0.38) n = 34	0.13 (0.41) n = 34	*0.147*
	TSH [μU/mL]°	1.8 (±0.8) n = 38	1.9 (±0.9) n = 39	*0.372*
	T3 [pg/mL]°	3.37 (±0.32) n = 39	3.31 (±0.39) n = 39	*0.476*
	T4 [ng/dL]^	1.30 (0.20) n = 39	1.27 (0.20) n = 39	*0.163*
	BMI [kg/m^2^]^	22.5 (3.6) n = 40	24.9 (5.9) n = 40	0.023*
	LBM [%]^	79.4 (7.0) n = 40	80.9 (9.0) n = 38	*0.838*
*Biomarkers of carbohydrate metabolism*	Glucose [mg/dL]°	81 (7) n = 39	86 (11) n = 40	0.016*
	HbA1c [%]^	5.0 (0.4) n = 40	5.1 (0.5) n = 40	*0.150*
	C-Peptide [ng/mL]^	1.2 (0.7) n = 39	1.3 (1.1) n = 39	0.009*
	Insulin [μU/mL]^	3.9 (2.4) n = 36	6.1 (5.8) n = 39	0.001*
*Biomarkers of lipid metabolism*	TChol [mg/dL]^	165 (49) n = 39	180 (71) n = 40	*0.344*
	HDL Chol [mg/dL]°	58 (±15) n = 39	57 (±14) n = 40	*0.761*
	LDL Chol [mg/dL]^	94 (36) n = 39	109 (70) n = 40	*0.410*
	LDL/HDL ratio^	1.6 (1.0) n = 39	1.8 (1.3) n = 40	*0.315*
	TG [mg/dL]^	73 (31) n = 38	95 (56) n = 40	0.023*
	LPA2 [mg/dL]^	22 (27) n = 37	14 (21) n = 37	0.048*
	ApoA1 [mg/dL]°	140 (±24) n = 40	141 (±24) n = 40	*0.882*
	ApoB [mg/dL]^	84 (33) n = 40	82 (57) n = 40	*0.939*
	Apo B/A1 ratio^	0.6 (0.2) n = 39	0.6 (0.3) n = 40	*0.521*

(Table 3 summarises and compares (GS versus C) biomarkers of energy-, carbohydrate and lipid metabolism of male subjects of the BiliHealth study.

Values are specified as applies according to distribution of data. For parametric variables, means° (±sd) are shown, for non-parametric data, medians^ (50^th^ percentiles) and inter-quartile range (IQR) are displayed. Comparison of means (for parametric data) or of ranks (for non-parametric data) was completed using independent samples t-test or Mann-Whitney-U-test (*p-value: significant on a 5% level of significance; ^T^p-value: trend on a 10% level of trend).

Abbreviations: GS: Gilbert’s syndrome; C: Controls; pAMPK α1/α2: Phosphorylated 5′-AMP activated kinase; pPpar α: Phosphorylated peroxisome proliferator activated receptor alpha; pPpar γ: Phosphorylated peroxisome proliferator activated receptor gamma; PgC 1α: Peroxisome proliferator-activated receptor c coactivator 1; AMPK α1 expr.: AMPK gene expression as relative quantification, RQ to cDNA pool; Sirt-1: Sirtuin-1; FGF-21: Fibroblast growth factor 21; TSH: Thyroid stimulating hormone; T3: Free triiodothyronine; T4: Free thyroxine; BMI: Body mass index; LBM: Lean body mass; HbA1c: Glycated haemoglobin A1c; TChol: Total cholesterol; HDL: High density lipoprotein cholesterol; LDL: Low density lipoprotein cholesterol; TG: Triglyceride; LPA2: Lipoprotein A2; ApoA1: Apolipoprotein A1; ApoB: Apolipoprotein B.

**Table 4:** Energy-, carbohydrate and lipid metabolic biomarkers of female subjects of the BiliHealth study.

FEMALES	Variable	Mean° (±sd)/median^ (IQR)		p-value^*/T^
		*GS*	*C*	
*Biomarkers of energy metabolism*	pAMPK α1/α2 [rfU]°	173 (±59) n = 19	135 (±49) n = 19	0.037*
	pPpar α [rfU]°	431 (±90) n = 19	283 (±58) n = 19	0.000*
	pPpar γ [rfU]°	1543 (±351) n = 19	1069 (±376) n = 19	0.000*
	PgC 1α [rfU]°	221 (±41) n = 19	167 (±33) n = 19	0.000*
	AMPK α1 expr. [RQ]^	0.81 (0.4) n = 20	0.85 (0.35) n = 20	*0.303*
	Sirt-1 [ng/mL]^	2.79 (1.74) n = 16	2.67 (0.96) n = 18	*0.731*
	FGF-21 [μg/mL]^	0.46 (0.65) n = 18	0.25 (0.67) n = 17	*0.428*
	TSH [μU/mL]^	1.9 (1.6) n = 19	2.1 (1.2) n = 20	*0.747*
	T3 [pg/mL]°	2.96 (±0.45) n = 19	3.04 (±0.35) n = 20	*0.530*
	T4 [ng/dL]°	1.25 (±0.15) n = 19	1.20 (±0.17) n = 20	*0.371*
	BMI [kg/m^2^]°	21.8 (±2.7) n = 20	24.7 (±4.4) n = 20	0.017*
	LBM [%]°	75.6 (±7.0) n = 19	69.2 (±7.8) n = 20	0.011*
*Biomarkers of carbohydrate metabolism*	Glucose [mg/dL]^	81 (8) n = 20	85 (10) n = 20	*0.136*
	HbA1c [%]°	5.0 (±0.5) n = 20	5.1 (±0.4) n = 20	*0.379*
	C-Peptide [ng/mL]^	1.3 (0.5) n = 19	1.5 (0.8) n = 20	*0.146*
	Insulin [μU/mL]^	4.3 (2.6) n = 19	4.7 (4.9) n = 20	*0.261*
*Biomarkers of lipid metabolism*	TChol [mg/dL]°	179 (±33) n = 19	194 (±33) n = 20	*0.154*
	HDL Chol [mg/dL]°	81 (±19) n = 19	76 (±20) n = 20	*0.476*
	LDL Chol [mg/dL]°	84 (±32) n = 19	103 (±31) n = 20	*0.071*^*T*^
	LDL/HDL ratio°	1.1 (±0.6) n = 19	1.5 (±0.7) n = 20	*0.099*^*T*^
	TG [mg/dL]^	70 (34) n = 19	66 (33) n = 20	*0.725*
	LPA2 [mg/dL]^	14.0 (12.0) n = 17	10.5 (17.0) n = 18	*0.865*
	ApoA1 [mg/dL]°	168 (±23) n = 20	174 (±28) n = 20	*0.485*
	ApoB [mg/dL]°	77 (±21) n = 20	89 (±23) n = 20	*0.087*^*T*^
	Apo B/A1 ratio^	0.5 (0.2) n = 20	0.5 (0.2) n = 20	*0.463*

(Table 4 summarises and compares (GS versus C) biomarkers of energy-, carbohydrate and lipid metabolism of female subjects of the BiliHealth study.

Values are specified as applies according to distribution of data. For parametric variables, means° (±sd) are shown, for non-parametric data, medians^ (50^th^ percentiles) and inter-quartile range (IQR) are displayed. Comparison of means (for parametric data) or of ranks (for non-parametric data) was completed using independent samples t-test or Mann-Whitney-U-test (*p-value: significant on a 5% level of significance; ^T^p-value: trend on a 10% level of trend).

Abbreviations: GS: Gilbert’s syndrome; C: Controls; pAMPK α1/α2: Phosphorylated 5′-AMP activated kinase; pPpar α: Phosphorylated peroxisome proliferator activated receptor alpha; pPpar γ: Phosphorylated peroxisome proliferator activated receptor gamma; PgC 1α: Peroxisome proliferator-activated receptor c coactivator 1; AMPK α1 expr.: AMPK gene expression as relative quantification, RQ to cDNA pool; Sirt-1: Sirtuin-1; FGF-21: Fibroblast growth factor 21; TSH: Thyroid stimulating hormone; T3: Free triiodothyronine; T4: Free thyroxine; BMI: Body mass index; LBM: Lean body mass; HbA1c: Glycated haemoglobin A1c; TChol: Total cholesterol; HDL: High density lipoprotein cholesterol; LDL: Low density lipoprotein cholesterol; TG: Triglyceride; LPA2: Lipoprotein A2; ApoA1: Apolipoprotein A1; ApoB: Apolipoprotein B.

**Table 5:** Stepwise linear regression analysis for key variables included in Fig. 2.

Dependent variables:	*pAMPK α1/α2*	*PgC1 α*	*Ppar α*	*Ppar γ*	*AMPK α1 expr.*	*UCB*	*UGT1A1 genotype*	*BMI*	*LBM*	*T3*
*pAMPK α1/α2*						0.190 (0.000)				
*PgC 1α*	0.070 ^GS^ (0.006)		0.743 (0.000)	0.781 (+Ppar α) (0.000)	0.060 (0.041)				0.083 ^GS^ (0.020)	
*Ppar α*		0.743 (0.000)					0.771 (+PgC1 α) (0.000)			
*Ppar γ*		0.496 (0.000)								
*AMPK α1 expr.*										
*BMI*						0.072 (0.004)				
*LBM*								0.483 (0.000)		0.538 (+BMI) (0.000)
*Sirt-1*									0.069 (0.009)	

Corrected R^2^ coefficients and corresponding p-values (in brackets) from stepwise linear regression analysis are provided.

Unspecified regressions are valid for the entire study population. ^C^effect valid for control subjects only; ^GS^effect valid for GS subjects only.

Abbreviations: pAMPK α1/α2: Phosphorylated 5′-AMP activated kinase; PgC 1α: Peroxisome proliferator-activated receptor c coactivator 1; pPpar α: Phosphorylated peroxisome proliferator activated receptor alpha; Ppar γ: Phosphorylated peroxisome proliferator activated receptor gamma; AMPK α1 expr.: AMPK α1 gene expression; UCB: unconjugated bilirubin; UGT1A1: UDP glucuronosyltransferase 1A1 polymorphism; BMI: Body mass index; LBM: Lean body mass; Sirt-1: Sirtuin-1; T3: Free triiodothyronine.

Included variables:

**pAMPK α1/α2:** AMPK1a expr., PgC1 α, pPpar α, pPpar γ, TChol, TG, UCB, UGT1A1 genotype, BMI, LBM, LDL/HDL, TSH, T3, T4.

**PgC 1 α:** pAMPK α1/α2, AMPK1a expr., Ppar α, Ppar γ, TChol, TG, UCB, UGT1A1 genotype, BMI, LBM, LDL/HDL, TSH, T3, T4.

**pPpar α:** pAMPK α1/α2, AMPK1a expr., Ppar γ, TChol, TG, UCB, UGT1A1 genotype, BMI, LBM, LDL/HDL, TSH, T3, T4.

**AMPK α1 expr.:** pAMPK α1/α2, Ppar α, Ppar γ, TChol, TG, UCB, UGT1A1 genotype, BMI, LBM, LDL/HDL, TSH, T3, T4.

**BMI:** AMPK α1 expr., pAMPK α1/α2, PgC1 α, Ppar α, Ppar γ, UCB, UGT1A1 genotype, LBM, TSH, T3, T4.

**LBM:** AMPK α1 expr., pAMPK α1/α2, PgC1 α, Ppar α, Ppar γ, UCB, UGT1A1 genotype, BMI, TSH, T3, T4.

**Sirt-1:** AMPK α1 expr., pAMPK α1/α2, PgC1 α, Ppar α, Ppar γ, UCB, UGT1A1 genotype, BMI, LBM, TSH, T3, T4, FGF21, TChol, TG, LDL/HDL.

(In further analyses the variables age, gender and those specifying lifestyle were included, however these procedures did not significantly change the models’ outcome).

**Table 6:** Stepwise linear regression analysis with reference to metabolic pathways (Fig. 2).

Dependent variables:	*C-Peptide*	*Glucose*	*TChol*	*TG*	*UCB*	*pPpar γ*	*LBM*	*BMI*
*HbA1c*		0.314 (0.000)				0.340 (+Glucose) (0.000)		
*C-Peptide*		0.575 (+TG, BMI) (0.000)		0.447 (0.000)				0.544 (+TG) (0.000)
*Insulin*				0.350 (0.000)				0.415 (+TG) (0.000)
*Glucose*	0.264 (0.000)							
*TChol*				0.158 (0.000)			0.205 (+TG) (0.000)	
*HDL*				0.187 (0.000)				
*LDL*				0.198 (0.000)				0.226 (+TG) (0.000)
*TG*			0.318 (+BMI) (0.000)					0.249 (0.000)
*Apo A1*							0.190 (+BMI) (0.000)	0.047 (0.018)
*Apo B*				0.274 (0.000)				0.303 (+TG) (0.000)
*LPA2*					0.034 (0.043)			

Corrected R^2^ coefficients and p-values (in brackets) from stepwise linear regression analysis, involving the entire study population, are provided.

HbA1c: Glycated haemoglobin A1c; pPpar γ: Phosphorylated peroxisome proliferator activated receptor gamma; T3: Free triiodothyronine; T4: Free thyroxine; BMI: Body mass index; LBM: Lean body mass; TChol: Total cholesterol; HDL: High density lipoprotein cholesterol; LDL: Low density lipoprotein cholesterol; TG: Triglyceride; LPA2: Lipoprotein A2; ApoA1: Apolipoprotein A1; ApoB: Apolipoprotein B.

Included variables:

**HbA1c:** UCB, TSH, T3, T4, UGT1A1-genotype, pAMPK α1/α2, PgC1 α, pPpar α, pPparγ, AMPK α1 expr., BMI, LBM, TG, FGF-21, C-Peptide, Glucose.

**C-Peptide/Insulin:** UCB, TSH, T3, T4, UGT1A1-genotype, pAMPK α1/α2, pPpar α, pPpar γ, PgC1 α, AMPK α1 expr., BMI, LBM, TG, FGF-21, Glucose, HbA1c.

**Glucose:** UCB, TSH, T3, T4, UGT1A1-genotype, pAMPK α1/α2, pPpar α, pPpar γ, PgC1 α, AMPK α1 expr., BMI, LBM, TG, FGF-21, C-Peptide, TChol, LDL/HDL.

**TChol:** UCB, TSH, T3, T4, UGT1A1-genotype, pAMPK α1/α2, pPpar α, pPpar γ, PgC1 α, AMPK α1 expr., BMI, LBM, TG, FGF-21.

**HDL:** UCB, TSH, T3, T4, UGT1A1-genotype, pAMPK α1/α2, pPpar α, pPpar γ, PgC1 α, AMPK α1expr., BMI, LBM, TG, FGF-21.

**LDL:** UCB, TSH, T3, T4, UGT1A1-genotype, pAMPK α1/α2, pPpar α, pPpar γ, PgC1 α, AMPK α1 expr., BMI, LBM, TG, FGF-21.

**TG:** UCB, TSH, T3, T4, UGT1A1-genotype, pAMPK α1/α2, pPpar α, pPpar γ, PgC1 α, AMPK α1 expr., BMI, LBM, TG, FGF-21, TChol, Glucose, HbA1c.

**Apo A1:** UCB, TSH, T3, T4, UGT1A1-genotype, pAMPK α1/α2, pPpar α, pPpar γ, PgC1 α, AMPK α1 expr., BMI, LBM, TG, FGF-21.

**Apo B:** UCB, TSH, T3, T4, UGT1A1-genotype, pAMPK α1/α2, pPpar α, pPpar γ, PgC1 α, AMPK α1 expr., BMI, LBM, TG, FGF-21.

**LPA2:** UCB, TSH, T3, T4, UGT1A1-genotype, pAMPK α1/α2, pPpar α, pPpar γ, PgC1 α, AMPK α1 expr., BMI, LBM, TG, FGF-21.

(In further analyses the variables age, gender and those specifying lifestyle were included, however these procedures did not significantly change the models’ outcome).

## References

[b1] Cantó C. & Auwerx J. PGC-1alpha, SIRT1 and AMPK, an energy sensing network that controls energy expenditure. Curr Opin Lipidol 20, 98–105 (2009). 19276888 10.1097/MOL.0b013e328328d0a4 PMC3627054

[b2] Lage R., Diéguez C., Vidal-Puig A. & López M. AMPK: a metabolic gauge regulating whole-body energy homeostasis. Trends Mol Med 14, 539–549 (2015). 18977694 10.1016/j.molmed.2008.09.007

[b3] Iyer L. . UGT1A1*28 polymorphism as a determinant of irinotecan disposition and toxicity. Pharmacogenomics J 2, 43–47 (2002). 11990381 10.1038/sj.tpj.6500072

[b4] Wallner M. . Haem catabolism: a novel modulator of inflammation in Gilbert’s syndrome. Eur J Clin Invest 43, 912–919 (2013). 23865893 10.1111/eci.12120

[b5] Wallner M. . Protection from age-related increase in lipid biomarkers and inflammation contributes to cardiovascular protection in Gilbert’s syndrome. Clin Sci 125, 257–264 (2013). 23566065 10.1042/CS20120661

[b6] Bulmer A. C., Verkade H. J. & Wagner K.-H. Bilirubin and beyond: A review of lipid status in Gilbert’s syndrome and its relevance to cardiovascular disease protection. Prog Lipid Res 52, 193–205 (2013). 23201182 10.1016/j.plipres.2012.11.001

[b7] Wagner K.-H. . Looking to the horizon: the role of bilirubin in the development and prevention of age-related chronic diseases. Clin Sci 129, 1–25 (2015). 25881719 10.1042/CS20140566

[b8] Vítek L. The role of bilirubin in diabetes, metabolic syndrome, and cardiovascular diseases. *role bile Pigment Heal Dis Eff cell signaling, Cytotox Cytoprot* 192 (2012). 10.3389/fphar.2012.00055 PMC3318228 22493581

[b9] Hardie D. G., Schaffer B. E. & Brunet A. AMPK: An Energy-Sensing Pathway with Multiple Inputs and Outputs. Trends Cell Biol, 10.1016/j.tcb.2015.10.013 (2016). PMC5881568 26616193

[b10] Hardie D. G. & Carling D. The AMP-Activated Protein Kinase. Fuel Gauge of the Mammalian Cell? Eur J Biochem Rev 246h, 259–273 (1997). 10.1111/j.1432-1033.1997.00259.x 9208914

[b11] Hardie D. G. AMP-activated protein kinase as a drug target. Annu Rev Pharmacol Toxicol 47, 185–210 (2007). 16879084 10.1146/annurev.pharmtox.47.120505.105304

[b12] Hawley S. A. . Characterization of the AMP-activated protein kinase kinase from rat liver and identification of threonine 172 as the major site at which it phosphorylates AMP-activated protein kinase. J Biol Chem 271, 27879–27887 (1996). 8910387 10.1074/jbc.271.44.27879

[b13] Zhang B. B., Zhou G. & Li C. AMPK: An Emerging Drug Target for Diabetes and the Metabolic Syndrome. Cell Metab 9, 407–416 (2009). 19416711 10.1016/j.cmet.2009.03.012

[b14] Fryer L., Parbu-Patel A. & Carling D. The Anti-diabetic Drugs Rosiglitazone and Metformin Stimulate AMP-activated Protein Kinase through Distinct Signaling Pathways. J Biol Chem 277, 25226–25232 (2002). 11994296 10.1074/jbc.M202489200

[b15] Handschin C. & Spiegelman B. M. Peroxisome Proliferator-Activated Receptor γ Coactivator 1 Coactivators, Energy Homewostasis, and Metabolism. Endocr Rev 27, 728–735 (2006). 17018837 10.1210/er.2006-0037

[b16] Coste A. . The genetic ablation of SRC-3 protects against obesity and improves insulin sensitivity by reducing the acetylation of PGC-1{alpha}. Proc Natl Acad Sci USA 105, 17187–17192 (2008). 18957541 10.1073/pnas.0808207105 PMC2579399

[b17] Issemann I. & Green S. Activation of a member of the steroid hormone receptor superfamily by peroxisome proliferators. Lett To Nat 346, 183–187 (1990). 10.1038/347645a0 2129546

[b18] Diradourian C., Girard J. & Pégorier J.-P. Phosphorylation of PPARs: from molecular characterization to physiological relevance. Biochimie 87, 33–38 (2005). 15733734 10.1016/j.biochi.2004.11.010

[b19] Michalik L. . International Union of Pharmacology. LXI. Peroxisome Proliferator-Activated Receptors. Pharmacol Rev 58, 726–741 (2006). 17132851 10.1124/pr.58.4.5

[b20] Pawar A. & Jump D. B. Unsaturated fatty acid regulation of peroxisome proliferator-activated receptor alpha activity in rat primary hepatocytes. J Biol Chem 278, 35931–35939 (2003). 12853447 10.1074/jbc.M306238200

[b21] Ricote M., Huang J. T., Welch J. S. & Glass C. K. The peroxisome proliferator-activated receptor (PPARgamma) as a regulator of monocyte/macrophage function. J Leukoc Biol 66, 733–739 (1999). 10577502 10.1002/jlb.66.5.733

[b22] Barish G. D., Narkar V. a. & Evans R. M. PPARδ: a dagger in the heart of the metabolic syndrome. J Clin Invest 116, 590–597 (2006). 16511591 10.1172/JCI27955 PMC1386117

[b23] Poulsen L. L. C., Siersbæk M. & Mandrup S. PPARs: Fatty acid sensors controlling metabolism. Semin Cell Dev Biol 23, 631–639 (2012). 22273692 10.1016/j.semcdb.2012.01.003

[b24] Tontonoz P. & Spiegelman B. M. Fat and Beyond: The Diverse Biology of PPARγ. Annu Rev Biochem 77, 289–312 (2008). 18518822 10.1146/annurev.biochem.77.061307.091829

[b25] Evans R. M., Barish G. D. & Wang Y.-X. PPARs and the complex journey to obesity. Nat Med 10, 355–361 (2004). 15057233 10.1038/nm1025

[b26] Liu J. . Bilirubin Increases Insulin Sensitivity by Regulating Cholesterol Metabolism, Adipokines and PPARγ Levels. Sci Rep 5, 9886 (2015). 26017184 10.1038/srep09886 PMC4446899

[b27] Vacca M., Degirolamo C., Mariani-Costantini R., Palasciano G. & Moschetta A. Lipid-sensing nuclear receptors in the pathophysiology and treatment of the metabolic syndrome. Wiley Interdiscip Rev Syst Biol Med 3, 562–587 (2011). 21755605 10.1002/wsbm.137

[b28] Woo Y. C., Xu A., Wang Y. & Lam K. S. L. Fibroblast Growth Factor 21 as an emerging metabolic regulator: Clinical perspectives. Clin Endocrinol (Oxf) 78, 489–496 (2013). 23134073 10.1111/cen.12095

[b29] Xu J. . Acute glucose-lowering and insulin-sensitizing action of FGF21 in insulin-resistant mouse models–association with liver and adipose tissue effects. Am J Physiol Endocrinol Metab 297, E1105–E1114 (2009). 19706786 10.1152/ajpendo.00348.2009

[b30] Kharitonenkov A. . The metabolic state of diabetic monkeys is regulated by fibroblast growth factor-21. Endocrinology 148, 774–781 (2007). 17068132 10.1210/en.2006-1168

[b31] Bigo C. . PPARα: A Master Regulator of Bilirubin Homeostasis. PPAR Res 2014, 747014 (2014). 25147562 10.1155/2014/747014 PMC4134828

[b32] Stocker R. Antioxidant activities of bile pigments. Antioxid Redox Signal 6, 841–849 (2004). 15345144 10.1089/ars.2004.6.841

[b33] Brito M. a. *et al*. Bilirubin injury to neurons: Contribution of oxidative stress and rescue by glycoursodeoxycholic acid. Neurotoxicology 29, 259–269 (2008). 18164405 10.1016/j.neuro.2007.11.002

[b34] Danoff T. . A Gilbert’s syndrome UGT1A1 variant confers susceptibility to tranilast-induced hyperbilirubinemia. Pharmacogenomics J 4, 49–53 (2004). 14647407 10.1038/sj.tpj.6500221

[b35] Bosma P. J. . The genetic basis of the reduced expression of bilirubin UDP-glucuronosyltransferase 1 in Gilbert’s syndrome. N Engl J Med 333, 1171–1175 (1995). 7565971 10.1056/NEJM199511023331802

[b36] Tosevska A. . Longer telomeres in chronic, moderate, unconjugated hyperbilirubinaemia: insights from a human study on Gilbert’s Syndrome. Sci Rep 6, 22300 (2016). 26926838 10.1038/srep22300 PMC4772088

[b37] Friedrichsen M., Mortensen B., Pehmøller C., Birk J. B. & Wojtaszewski J. F. P. Exercise-induced AMPK activity in skeletal muscle: Role in glucose uptake and insulin sensitivity. Mol Cell Endocrinol 366, 204–214 (2013). 22796442 10.1016/j.mce.2012.06.013

[b38] O’Neill L. A. J. & Hardie D. G. Metabolism of inflammation limited by AMPK and pseudo-starvation. Nature 493, 346–355 (2013). 23325217 10.1038/nature11862

[b39] Yamauchi T. . The Mechanisms by Which Both Heterozygous Peroxisome Proliferator-activated Receptor gamma (PPAR-gamma) Deficiency and PPAR-gamma Agonist Improve Insulin Resistance. J Biol 276, 41245–41254 (2001). 10.1074/jbc.M103241200 11533050

[b40] Guan H.-P. . A futile metabolic cycle activated in adipocytes by antidiabetic agents. Nat Med 8, 1122–1128 (2002). 12357248 10.1038/nm780

[b41] Sato R., Goldstein J. & Brown M. Replacement of serine-871 of hamster 3-hydroxy-3-methylglutaryl-CoA reductase prevents phosphorylation by AMP-activated kinase and blocks inhibition of sterol synthesis induced by ATP depletion. Proc Natl Acad Sci USA 90, 9261–9265 (1993). 8415689 10.1073/pnas.90.20.9261 PMC47547

[b42] Dong H. . Bilirubin increases insulin sensitivity in leptin-receptor deficient and diet-induced obese mice through suppression of ER stress and chronic inflammation. Endocrinology 155, 818–828 (2014). 24424052 10.1210/en.2013-1667 PMC3929745

[b43] Chu N.-F. . Plasma insulin, leptin, and soluble TNF receptors levels in relation to obesity-related atherogenic and thrombogenic cardiovascular disease risk factors among men. Atherosclerosis 157, 495–503 (2001). 11472752 10.1016/s0021-9150(00)00755-3

[b44] Kahn B. B. & Flier J. S. Obesity and insulin resistance. J Clin Invest 106, 473–481 (2000). 10953022 10.1172/JCI10842 PMC380258

[b45] Li Y. . Extracellular Nampt Promotes Macrophage Survival via a Nonenzymatic Interleukin-6/STAT3 Signaling Mechanism. J Biol Chem 283, 34833–34843 (2008). 18945671 10.1074/jbc.M805866200 PMC2596403

[b46] Tamrakar P., Ibrahim B. A., Gujar A. D. & Briski K. P. Estrogen regulates energy metabolic pathway and upstream adenosine 5′-monophosphate-activated protein kinase and phosphatase enzyme expression in dorsal vagal complex metabolosensory neurons during glucostasis and hypoglycemia. J Neurosci Res 93, 321–332 (2015). 25231731 10.1002/jnr.23481 PMC4270942

[b47] Rogers N. H., Witczak C. A., Hirshman M. F., Goodyear L. J. & Greenberg A. S. Estradiol stimulates Akt, AMP-activated protein kinase (AMPK) and TBC1D1/4, but not glucose uptake in rat soleus. Biochem Biophys Res Commun 382, 646–650 (2009). 19265681 10.1016/j.bbrc.2009.02.154 PMC2692044

[b48] Wallner M. . Anti-Genotoxic Potential of Bilirubin *In Vivo*: Damage to DNA in Hyperbilirubinemic Human and Animal Models. Cancer Prev Res 6, 1056–1063 (2013). 10.1158/1940-6207.CAPR-13-0125 23983086

[b49] Radu P. & Atsmon J. Gilbert’s syndrome-clinical and pharmacological emplications. Isr Med Assoc J 3, 593–598 (2001). 11519385

[b50] Brower J. O., Lightner D. a. & McDonagh A. F. Aromatic congeners of bilirubin: synthesis, stereochemistry, glucuronidation and hepatic transport. Tetrahedron 57, 7813–7827 (2001).

[b51] Mölzer C. . *In vitro* antioxidant capacity and antigenotoxic properties of protoporphyrin and structurally related tetrapyrroles. Free Radic Res 46, 1369–1377 (2012). 22861140 10.3109/10715762.2012.715371

[b52] Bisht K. . Endogenous Tetrapyrroles Influence Leukocyte Responses to Lipopolysaccharide in Human Blood: Pre-Clinical Evidence Demonstrating the Anti-Inflammatory Potential of Biliverdin. J Clin Cell Immunol 5, 1000218 (2014). 25177524 10.4172/2155-9899.1000218 PMC4145741

[b53] Von Ahsen N., Oellerich M. & Schutz E. DNA base bulge vs unmatched end formation in probe-based diagnostic insertion/deletion genotyping: Genotyping the UGT1A1 (TA)(n) polymorphism by real-time fluorescence PCR. Clin Chem 46, 1939–1945 (2000). 11106326

[b54] Roubenoff R. . Application of bioelectrical impedance analysis to elderly populations. J Gerontol Med Sci 52A, M129–M136 (1997). 10.1093/gerona/52a.3.m129 9158553

