# Supplementary material for: Features of an altered AMPK metabolic pathway in Gilbert’s Syndrome, and its role in metabolic health
Source: Sci Rep. 2016 Jul 21;6:30051. doi: 10.1038/srep30051 (PMC4956769; doi:10.1038/srep30051)
Supplement: Supplementary Information [file srep30051-s1.pdf]

"Features of an altered AMPK metabolic pathway in Gilbert's Syndrome, and its role in energy turnover and metabolic health."

Christine Mölzer, Marlies Wallner, Carina Kern, Anela Tosevska, Ursula Schwarz, Rene Zadnikar, Daniel Doberer, Rodrig Marculescu, Karl-Heinz Wagner

SUPPLEMENTARY FIGURES:

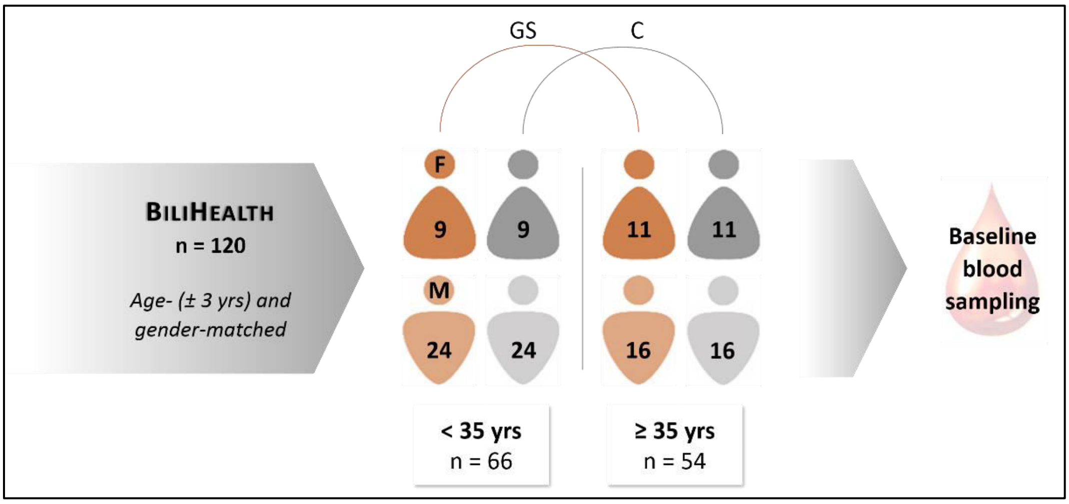

Supplementary figure S1. **BiliHealth study design.** A total of 80 males and 40 females (n = 120) completed the study. Subjects were age- and gender-matched. Study group allocation (Gilbert's Syndrome, GS; controls, C) was based on the subjects' respective serum UCB concentrations (unconjugated bilirubin </≥ 17.1 µM). Participants were furthermore allocated to age groups (</≥ 35 years of age). All subjects underwent a baseline blood sampling.

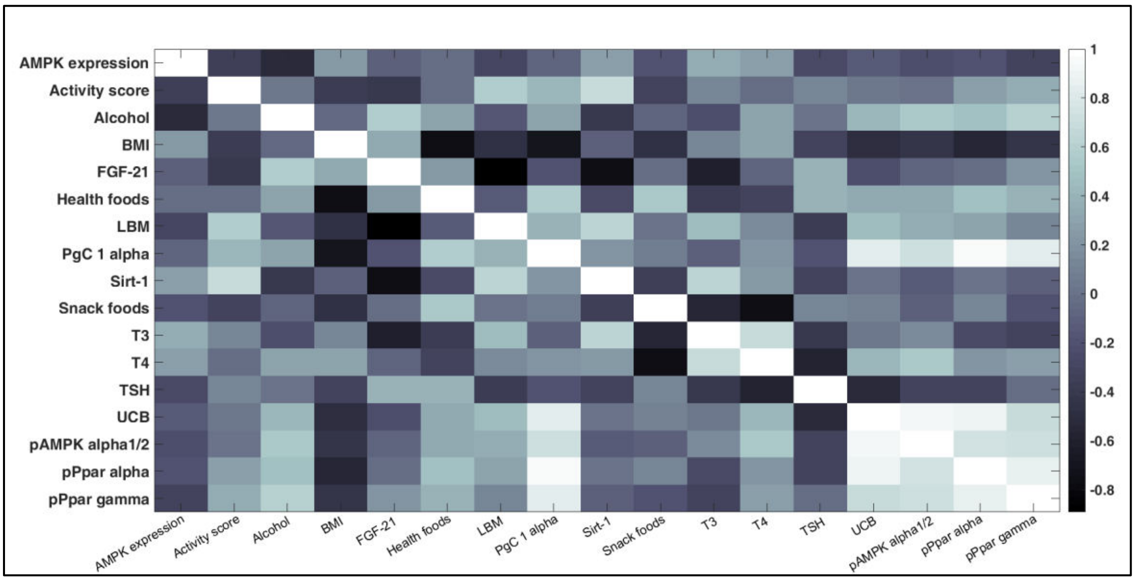

Supplementary figure S2 a. **Heat map showing inter-variable connection for markers of energy-metabolism.**

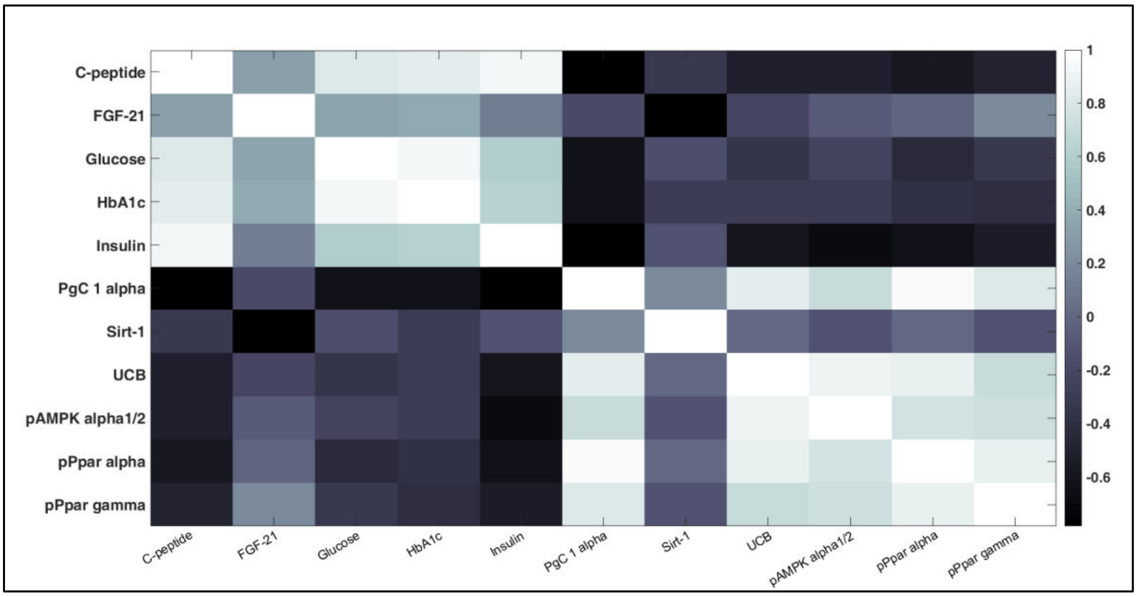

<u>Supplementary figure S2 b.</u> Heat map showing inter-variable connection for markers of glucose-metabolism.

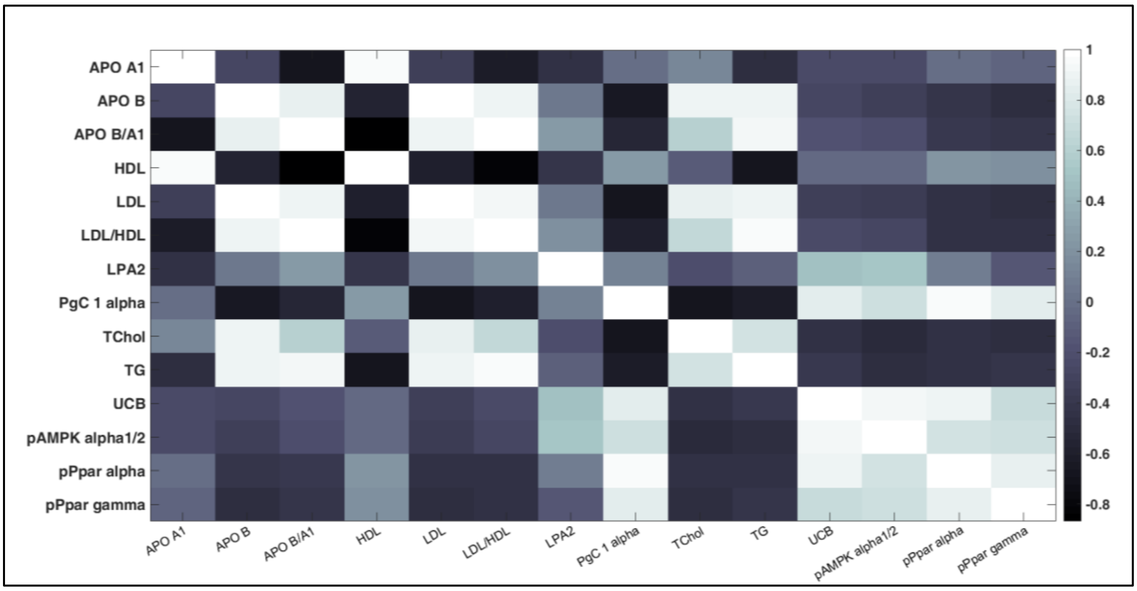

<u>Supplementary figure S2 c.</u> Heat map showing inter-variable connection for markers of lipid-metabolism.

<u>Supplementary figures S2 a – c.</u> Heat maps showing inter-variable connection with respect to energy- (a), glucose- (b) and lipid metabolism (c). Heat maps were generated using the COVAIN toolbox for MATLAB [MATLAB and Statistics Toolbox Release 2015a, The MathWorks, Inc., Natick, Massachusetts, United States.], and including the entire dataset (all subjects).

<u>Abbreviations:</u> UCB: unconjugated bilirubin; UGT1A1: UGT1A1 genotype; pAMPK α1/α2: Phosphorylated 5'-AMP activated kinase; pPpar α: Phosphorylated peroxisome proliferator activated receptor alpha; pPpar γ: Phosphorylated peroxisome proliferator activated receptor gamma; PgC 1α: Peroxisome proliferator-activated receptor c coactivator 1; AMPK1a expr.: AMPK gene expression as relative quantification, RQ to cDNA pool; Sirt-1: Sirtuin-1; FGF-21: Fibroblast growth factor 21; TSH: Thyroid stimulating hormone; T3: Free triiodothyronine; T4: Free thyroxine; BMI: Body mass index; LBM: Lean body mass; HbA1c: Glycated haemoglobin A1c; TChol: Total cholesterol; HDL: High density lipoprotein cholesterol; LDL: Low density lipoprotein cholesterol; TG: Triglyceride; LPA2: Lipoprotein A2; ApoA1: Apolipoprotein A1; ApoB: Apolipoprotein B; Activity score: self-reported daily activity (walking, climbing stairs, physical exercise); Alcohol/Health foods/Snack foods: self-reported weekly consumption.
